# Video Interventions for Reducing Health Inequity in Cancer Screening Programmes: a Systematic Review

**DOI:** 10.1007/s40615-023-01749-5

**Published:** 2023-08-21

**Authors:** Afua Richardson-Parry, Mitchell Silva, Jose Maria Valderas, Shaantanu Donde, Seth Woodruff, Joris van Vugt

**Affiliations:** 1 Viatris, Building 4, Trident Place, Mosquito Way, Hatfield, AL10 9UL UK; 2 Esperity, Veldkapelgaarde 30b1.30.30, 1200 Brussels, Belgium; 3 Department of Family Medicine, National University Health System and Yong Loo Lin School of Medicine, 1E Kent Ridge Road, NUHS Tower Block, Singapore, 119228 Singapore; 4 Viatris, Building 4, Trident Place, Mosquito Way, Hatfield, AL10 9UL UK; 5 Viatris, 235 E42nd Street, New York, NY 10017 USA; 6 Viatris, Krijgsman 20, Amstelveen, 1186DM The Netherlands

**Keywords:** Oncology, Screening, Colorectal cancer, Breast cancer, Cervical cancer, Mammography, Health equity, Health inequality, DVD

## Abstract

**Background:**

Health equity can lead to disparities in cancer screening, treatment, and mortality. This systematic review aims to identify and describe interventions that used video or DVD formats to reduce health inequity in cancer screening and review the effectiveness of such interventions in increasing screening rates compared to usual care conditions.

**Methods:**

We searched PubMed, Web of Science, Embase, and Cochrane databases for randomized control trials (RCTs) published until 18/01/2023 that compared intervention versus usual care control groups, with the percentage of cancer screening uptake during follow-up as an outcome. The risk of Bias was assessed with the Cochrane Collaboration tool.

**Results:**

After screening 4201 abstracts, 192 full texts were assessed for eligibility and 18 were included that focused on colorectal (*n* = 9), cervical (*n* = 5), breast (*n* = 5), and prostate (*n* = 1) cancer screening. All were based in the USA except one and most focused on ethnicity/race, while some included low-income populations. Most of the video interventions used to increase cervical cancer screening reported positive results. Studies aimed at increasing mammography uptake were mostly effective only in specific groups of participants, such as low-income or less-educated African American women. Results for colorectal cancer screening were conflicting. Videos that were culturally tailored or used emotive format were generally more effective than information-only videos.

**Conclusions:**

Video interventions to increase cancer screening among populations with low screening uptake show some positive effects, though results are mixed. Interventions that use individual and cultural tailoring of the educational material should be further developed and investigated outside of the USA.

## Introduction

The World Health Organization (WHO) defines health inequity as “systematic differences in the health status of different population groups” [https://www.who.int/news-room/facts-in-pictures/detail/healthinequities-and-their-causes]. Health inequity arises from many causes, including social, economic, environmental, and structural disparities that contribute to differences in health outcomes within and between societies. For many cancers, health inequity occurs at several levels, with differences observed in screening detection, diagnosis, treatment, and mortality. Screening at the public health and population level is an important strategy, especially for cervical, prostate, breast, and colorectal (CRC) cancers. For example, breast cancer mortality can be reduced with mammography screening [1] and CRC mortality through guaiac fecal occult blood testing (FOBT) or flexible sigmoidoscopy [2] screening techniques. A UK study reported that cervical cancer screening currently prevents 70% of cervical cancer deaths and estimated that 83% of such deaths could be prevented if everyone attended screening regularly [3].

Studies, mostly from the USA, have revealed lower cancer screening rates in certain racial and ethnic groups [4–6], immigrants [7], those with low-income or living in socio-economically deprived neighbourhoods [7, 8], and people living in rural areas [9]. The reasons behind differences in cancer screening uptake are complex and multifactorial. Sociodemographic and cultural norms [10, 11], as well as perceived susceptibility, benefits, and barriers, can all contribute to screening intention or completion [11]. Cancer stigma is significantly higher in men and in those from ethnic minority backgrounds and is associated with not being screened as recommended for cervical, breast, and colorectal cancer [12]. A review on cervical cancer screening identified numerous sociocultural factors influencing health-related beliefs and healthcare utilization among immigrant and ethnic minorities in the US [6] and the authors recommended that culturally relevant screening strategies should be developed to address growing health inequity [6]. Interventions that focus on social determinants of health to improve breast, cervical, and colorectal cancer screening appear to be cost-effective for underserved populations in the US because the increase in screening can lead to earlier diagnosis and treatment, better health outcomes, and improvements in quality-adjusted life-years [13]. Different interventions that focus on the barriers and motives underlying the lack of screening have been evaluated such as letters or alerts to remind people to attend a screening, using lay health workers or healthcare professionals to deliver group or individual health counselling and education, designing ethnically and culturally tailored print or video materials, providing financial incentives, and using interactive multimedia programs and decision aids [14–27]. Some trials have used video and DVDs to target groups with low cancer screening, to deliver information about the importance of screening, and different screening modalities and to address potential barriers to screening. These may be especially relevant now due to disruptions to routine screening services during the COVID-19 pandemic [28] as they have the potential to be delivered remotely. As results differ between studies, it will be useful to systematically evaluate the current evidence to provide an overview of how effective such interventions are for improving cancer screening, especially as they may be more cost-effective than some of the other more complex interventions.

Therefore, the objective of the current review is to describe interventions that used video or DVD formats to increase cancer screening in populations with low screening uptake and review the effectiveness of such interventions in increasing screening rates. Specifically, we focus on interventions that use videos or DVDs to deliver information with specific aims to i) assess whether participants who are shown video and DVD interventions aimed at increasing screening uptake have higher screening over follow-up than people receiving standard screening programs (usual care) and ii) compare different types of video delivery, for example, informative videos versus other types of video format (i.e., emotive videos that use a narrator who is a cancer survivor or culturally tailored storylines etc.).

## Method

The review was reported in accordance with the Preferred Reporting Items for Systematic Reviews and Meta-Analyses (PRISMA) recommendations [29]. In this review, we include papers covering various types of health inequity, including those related to ethnicity and race, low income, and low educational status. Due to heterogeneity between studies, we did not include a meta-analysis.

### Search Strategy and Selection Criteria

We searched four databases for articles published until 18/01/2023: 1) PubMed electronic database of the National Library of Medicine; 2) Web of Science; 3) Embase and; 4) Cochrane. Medical subject heading (MeSH) terms and free words referring to health equity and cancer screening were used as keywords. The PubMed search string is shown in Appendix 1.

References from the selected papers and from other relevant articles were also screened for potential studies. We used a PICOS to define relevant articles. *Population* included groups of people that are disproportionately affected by disparities, such as ethnic minorities and people with low income or educational levels. We focused only on cancers that are usually screened at the general population level as a public health strategy for everyone of a certain age (e.g., we did not include, for example, screening for lung cancer as it is not routinely done in people unless they are in high-risk groups such as heavy smokers etc.). Thus, we focused on CRC, breast, prostate, and cervical cancer. *Intervention* included any intervention to increase cancer screening uptake that used a video or DVD method to provide information to a specific group of people (low SES, ethnic minority groups etc.). *Comparison* was measured in two ways. First, we compared interventions versus usual care (i.e., usual screening invitation and process). Second, we compared different methods to deliver the information in the videos (for example, comparing culturally tailored videos to informative videos etc.). *Outcome* was a percentage of cancer screening uptake during follow-up (self-reported or medical record documentation of screening completion). Any type of screening was included, such as pap tests, HPV self-sampling test kits, mammographies, clinical breast exams, fecal immunochemical test (FIT), FOBT, flexible sigmoidoscopy, colonoscopy, etc.). *Study design* was limited exclusively to RCTs.

### Study Selection and Data Extraction

**Fig. 1:**
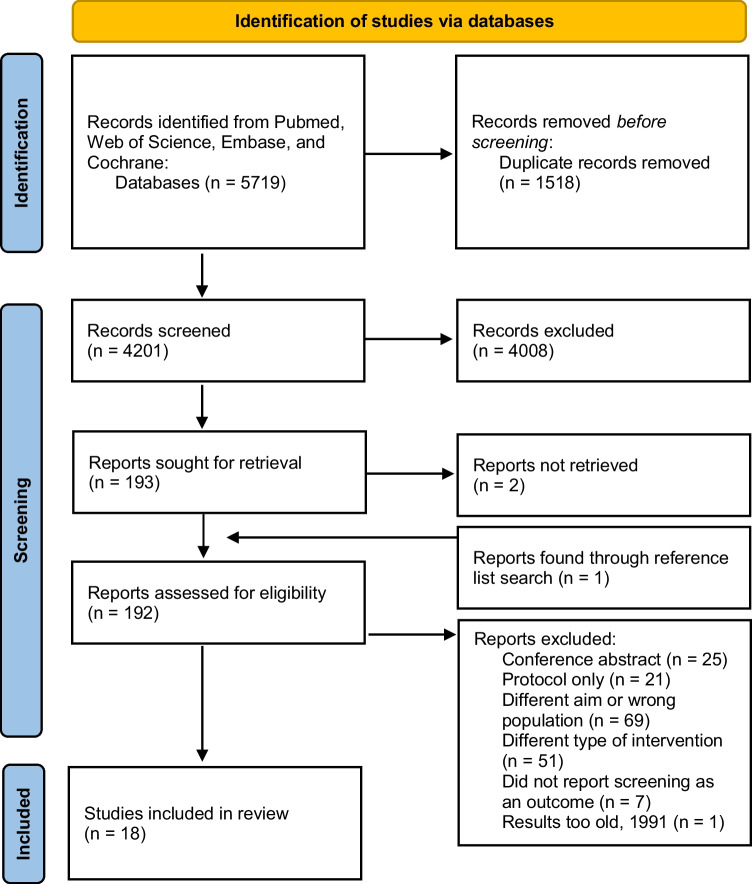
Identification of studies via databases

Two reviewers independently screened the titles and abstracts of the selected studies. Table 1 shows the exclusion criteria. The full texts of the articles selected by one or more of the reviewers were retrieved for evaluation. Two reviewers independently read the full texts and extracted the information from the selected studies. A third person reviewed the data extraction, and any disagreement was resolved through consensus. The numbers of abstracts screened, and studies assessed for eligibility, with reasons for exclusions at each stage, are presented in Fig. 1.

**Table 1:** Exclusion and inclusion criteria

The inclusion criteria were:
1) articles in English;
2) study design: randomized control trials;
3) usual care comparison group
Articles were excluded if they:
1) did not investigate the aims of the review;
2) did not report original data (e.g., < editorial) or was not peer-reviewed (e.g., congress abstract);
3) did not specifically examine ethnic minorities or people with low income or educational level;
4) had more complex types of intervention rather than just videos/DVDs (e.g., if a video was also used in conjunction with counselling or group education, it was excluded unless there was a video-only trial arm) and;
5) did not have a clear outcome related to screening uptake

### Data Extraction

Data extraction was conducted by one researcher and checked by another. Information was extracted on study design, number of participants (controls and intervention), participant demographics and baseline characteristics, type of cancer screening, type of intervention (including a description of the intervention), comparison group, and outcome (screening uptake). Data was recorded using RevMan.

### Assessment of Risk of Bias

The Review Manager software and the Cochrane Risk of Bias Tool were used for a methodological quality assessment of the risk of bias of the included studies [30]. The following domains were evaluated: (1) selection bias: sequence generation, allocation concealment; (2) detection bias: blinding of outcome assessment; (3) attrition bias: incomplete outcome data; and (4) reporting bias: selective reporting. In the case of a low possibility of bias, the studies were categorized as “low risk”, in the case of a high possibility of bias — “high risk” and if the occurrence of risk of bias could not be indicated — “unclear risk”. An in-detail summary of the risk of bias assessment is included in Appendix 2 (Tables 1 to 16). Bias assessment was done independently by two authors and discussed to reach a consensus in case of disagreement.

## Results

### Search Results

Figure 1 presents the results of the search; 4201 papers were identified in the search after duplicates were removed. After screening the titles and abstracts, 193 were assessed for eligibility but 2 reports could not be retrieved. One additional publication was found during the reference list search, but the data were from 1991 and it was excluded, as it is likely that the data are not relevant to the current health equity field. We excluded 25 conference abstracts and 21 protocols. After reading the full text, 18 were included in the final review [23, 31–47].

### Study Characteristics

The characteristics of the studies are shown in Tables 2–5. One study was conducted in New Zealand [32] and the remainder were from the USA. Most of the interventions were targeted to populations with either low socioeconomic status (low household income, high rates of unemployment, not covered by medical insurance) or to specific cultural or ethnic/racial populations in the USA, including Latino/Hispanic, Chinese women, and African American populations, as well as Maori and Pacific people in New Zealand. Although some studies had multiple outcomes, for example, change in health literacy or screening knowledge or beliefs, we only extracted data relevant to the aim of this review, namely screening completion. Follow-up times ranged from 4 weeks to 12 months, but most were 6 months.

**Table 2:** Cervical cancer screening: Study characteristics and results of screening completion (*n* = 5)

Author Year Country	Population N (Int, Cnt) Age range	Intervention description	Control	Results: Screening completion
Byrd USA, 2013[40]	Women of Mexican origins Int 1: 151 Int 2: 154 Int 3: 155 Cnt: 153 Age: mean 39.8 years	AMIGAS intervention, designed for delivery by trained lay health workers (promotoras de salud or promotoras) in either English or Spanish either in a group setting or individually. The intervention included a video that uses role modelling by women from the community to address common barriers and beliefs about cervical cancer and screening, a flip chart that reinforces the video and adds more in-depth information, and games and handouts that can be used by the promotora at her discretion to reinforce the messages in the video and flip chart Int 1: training by the promotora, an instructional video and a flip chart Int 2: video only Int 3: flip chart only	No promotora education but may have received education about cervical cancer screening delivered by clinics and the media	Self-reported cervical cancer screening Follow-up: 12 months Int 1: 52.3% Int 2: 41.3% Int 3: 45.5% Cnt: 24.8%, *p* < 0.001 There was no statistically significant difference among the 3 intervention arms
Calderon-Mora 2022, USA [41]	Latinas along the U.S.–Mexico border Int: *n* = 250 Cnt: *n* = 250 Age: 21–65	A novella-style video was adapted from the AMIGAS (Ayudando a las Mujeres con Información, Guía, y Amor para su Salud) program. 17-min video with info on the importance the pap test and how it is performed, followed by infographics and narration describing how cervical cancer develops, incidence and mortality rates, and risk factors	20-min flip chart presentation contained the same topics and information as the video	Self-reported pap-test Follow-up: not reported RR = 0.89 CI = 0.72–1.10, *p* = 0.294 For group age 51–65 RR = 1.44, CI = 1.08–1.92, *p* = 0.012
Rivers 2005, USA [42]	Low-income women Overall: *n* = 312 Subgroups not specified Age: 18–64	Group video. 10-min video that was gain-framed (i.e., the messages emphasise the gains and benefits of getting a Pap test) and 2 behaviour functions were assigned, i.e., emphasizing the i) prevention or ii) detection functions of the Pap test. E.g., “If you get regular Pap smears, you can prevent cervical cancer from developing... and preventing cervical cancer can save your life.”	Similar 10-min video with loss-framed messages “e.g., If you don’t get regular Pap smears, you can’t prevent cervical cancer from developing... and not preventing cervical cancer can cost your life.”	Self-reported screening Follow-up: 6 months Gain-framed + prevention:43.8% Loss-framed + prevention 40.5% Gain-framed + detection: 41.5% Loss-framed + detection: 51.9% When the Pap was described as a detection behaviour, odds for screening in loss-framed vs gain framed OR = 2.0; CI = 0.91–4.39 When the Pap was described as a prevention behaviour, odds for screening in loss-framed vs gain framed OR = 1.14, CI = 0.55–2.36
Taylor 2002, USA [43]	Chinese women in North America (Seattle and Vancouver) Int1: *n* = 129 Int2: *n* = 139 Cnt: *n* = 134 Age: 20–69	Chinese-language materials were used in both experimental arms: an education-entertainment video, a motivational pamphlet, an educational brochure, and a fact sheet Int1: outreach worker intervention. Participants received the materials, as well as tailored counselling and logistic assistance, during home visits by trilingual, bicultural outreach workers Int2: direct mail intervention. Participants received the materials by mail	Usual care	Self-reported cervical cancer screening Follow-up: 6 months Int1: 39% vs Cnt 15%, *p* < 0.001 Int2: 25% vs Cnt 15%, *p* = 0.03 Int1: 39% vs Int2: 25% *p* = 0.02
Thompson 2017, USA [48]	Rural Latina women Int1: *n *= 150 Int2: *n* = 146 Cnt: *n* = 147 Age: 21–64	Int1: low-intensity intervention arm – video in Spanish language, delivered to participants homes. Video based on a curriculum developed with community-based participatory research and social cognitive theory. Contained info about cervical cancer screening, encouragement to undergo screening, and info about low-cost clinics where women could go for the screening Int2: high-intensity intervention arm—video + home-based educational session led by a trained promotora	Usual care	Screening uptake (pap-test) Follow-up: 7 months Int1: 38.7% vs Cnt: 34.0%, *p* = 0.40 Int2: 53.4% vs Cnt: 34.0%, *p* < 0.001 Int2: 53.4% vs Int1: 38.7%, *p* < 0.01

CI = confidence interval; Cnt = control group; HPV = human papillomavirus; Int = Intervention group; NCI = National Cancer Institute; OR = odds ratio

### Video Interventions

The studies used videos or DVDs to provide information to participants about cancer screening. A range of information was included such as general information about cancer risks, risk factors, and the importance of screening, and often they showed films of the screening process. In many cases, the studies compared different modalities of information delivery, for example, comparing factual videos to emotive ones (e.g., featuring a cancer survivor), or added cultural tailoring (e.g., designed to debunk culturally based beliefs about cancer or screening, or using language and narrators of the same race or ethnicity as participants etc.). One [49] used an interactive DVD with 36 combinations of messages that changed according to belief questions that the participants answered using arrows on the DVD remote. According to the PICOS and exclusion criteria, we tried to include only studies that had only used videos alone, but some combined the video with another method, such as a brochure. We excluded studies where the video was only one part of a much larger intervention with multiple components. The comparison conditions were mostly usual care/normal screening but some compared video alone with combinations of other intervention components, for example, there were four arms in the ACCION study [38, 39], one which used a “promotora” (lay health advisor from the Hispanic community), one which used only a video to deliver information, and one that included both video and promotora, compared to a no-intervention control.

### Cervical Cancer Screening

Our search identified five studies on cervical cancer screening [40–44], as shown in Table 2. Calderon-Mora et al. [41] found no significant effect of their novella-style video on screening completion in Latina women compared to an information flipchart, but they did find an effect within women aged 51–65. Rivers et al. [42] demonstrated that the way that messages were delivered through video had an effect on screening rates but the effect differed on how they were worded. Although results did not reach statistical significance when pap tests were described as a detection behaviour, participants shown “loss-framed” messages (e.g., that emphasized negative aspects of what could happen if you do not get screening) had double the odds of completing screening than participants who watched gain-framed messages (e.g., “if you don’t get regular pap tests, you can’t detect cervical cancer early” versus “if you get regular pap tests, you can detect cervical cancer early”).

Byrd et al. [40] reported significantly higher self-reported cervical cancer screening in participants in the video-only arm of their study compared to controls but, interestingly, the screening rates did not differ in the video arm compared to a more complex intervention arm that included training by a promotora in conjunction with the instructional video. In contrast, a study on Chinese women in North America [43] found that an intervention containing an education-entertainment video, a motivational pamphlet, an educational brochure, and a fact sheet, increased screening completion (25–39%) compared to usual care (15%) but when the materials were delivered with an outreach worker who provided tailored counselling screening rates were significantly higher (39%) than when participants received the material by post (25%). Similarly, Thompson et al. [48] reported the same results in Latina women, though the Spanish language video was not statistically significantly better at increasing screening rates than usual care, there was only an effect when the video was present in combination with a home-based educational session led by a trained promotora. After a reference list search, we also identified a study from the UK of potential interest [50] on Asian women, but it was not included as it was published in 1991 and the results are unlikely to be relevant to the current field.

### Prostate Cancer Screening

There was only one study on prostate cancer screening [45] (Table 3), which used an intervention consisting of a 25-min videotape focusing on a middle-aged African American man as he discusses prostate cancer screening with his friends, family, and doctor. Participants in the intervention group did not have higher odds of prostate screening completion than controls.

**Table 3:** Prostate cancer screening: Study characteristics and results of screening completion (*n* = 1)

Author Year Country	Population N (Int, Cnt) Age range	Intervention description	Control	Results: Screening completion
Taylor 2006, USA [45]	African American men Int1: *n* = 80 Int2: *n* = 84 Cnt: *n* = 78 Age: 40–70	Int1: video-based information study arm. The 25-min videotape focuses on a middle-aged African American man as he discusses prostate cancer screening with his friends, family, and doctor Int2: print-based information study arm, 16-page, three-color, printed guide including prostate cancer symptoms, anatomy and function, prostate cancer risk factors, the benefits and limitations of screening, sample questions for men to ask their doctors, and a glossary of terms	Waiting list control study arm	Self-reported screening completion Follow-up: 12 months Direct rectal examination OR = 1.8, CI = 0.87–3.8 Prostate-Specific Antigen (PSA) test OR = 1.5, CI = 0.69–3.1

CI = confidence interval; Cnt = control group; Int = Intervention group; OR = odds ratio

### Breast Cancer Screening

We identified five studies that examined breast cancer screening as an outcome (Table 4). One [31] reported a small but not significant effect of a videotape on increasing mammography screening. Champion et al. [49] utilized an interactive DVD containing both video and other visual presentations to deliver tailored messages to participants. African American women with incomes below $75,000 who were in the interactive DVD group completed significantly more mammograms than women in usual care over follow-up. Similar results were reported by Gathirua-Mwangi et al. [46] in their study on African American women; for women with low incomes (≤ $30,000) a tailored narrative DVD intervention increased the odds of mammography five times compared to usual care, but no effect was seen in women with higher income levels. Kreuter et al. [23] also reported different success rates of their intervention depending on the characteristics of the patient. Specifically, in women with lower education (< 12 years) a narrative video format (with personal stories from African American breast cancer survivors) improved mammography completion compared to an informative, factual video narrated by an African American woman, but no effects were seen in women with higher educational levels (more than 12 years). In a trial on Chinese American immigrants [47] assessed acculturation, which was dichotomized according to English language ability and years of US residency. The culturally targeted video significantly increased mammography screening among low-acculturated women compared to the control condition (fact sheet).

**Table 4:** Breast cancer screening: Study characteristics and results of mammography completion (*n* = 5)

Author Year Country	Population N (Int, Cnt) Age range	Intervention description	Control	Results: Screening completion
Avis 2004, USA [31]	African American 31.7% Hispanic 23.1% Int: *n* = 900 Cnt: *n* = 900 Age: 50–75	Videotape, entitled ‘‘Mammograms for Life’’ and produced in both English and Spanish, aimed broadly at women over age 50 with a particular focus on minority women over age 60. 23 min long, contained documentary footage of interviews with women of diverse ethnic, geographic, and socioeconomic backgrounds, a leading medical expert in the field of breast oncology, and shows a woman getting a mammogram	2-page easy-to-read Pamphlet ‘‘Mammograms: Not Just Once, But For A Lifetime’’ distributed by the National Cancer Institute in both English and Spanish	Mammography completion Follow-up: 12 months Int: 80.4% vs. Cnt: 74.8% OR = 1.5, CI = 0.95–2.3
Champion 2016, USA [49]	African American 15.3% Differing income levels Int 1: *n* = 542 Int 2: *n* = 559 Cnt: *n* = 537 Age: 51–75	Int1: a mailed tailored interactive DVD. First tailored messages were developed for the theoretical constructs of perceived and actual risk, benefits, self-efficacy, and barriers as well as age and race. Tailoring was done by first asking participants to respond to a question about each belief. Participants responded to the belief questions using the arrows on the DVD remote. Message responses were selected based on algorithms built into the DVD program. There were 36 or more combinations of messaging. Video and other visual representations were used Int2: a computer-tailored telephone counselling. Developed using the same tailoring variables and messages used in the interactive DVD, keeping the message content consistent so that intervention arms varied only by media delivery	Usual care	Mammogram completion Follow-up: 6 months High income (> $75k) Int1 vs Cnt: OR = 0.6, CI = 0.4–0.97, *p* = 0.03 Int2 vs Cnt: OR = 1.1, CI = 0.7–1.7, *p* = 0.64 Low/middle income (< $75k) Int1 vs Cnt: OR = 1.5, CI = 1.1–2.1, *p* = 0.017 Int2 vs Cnt: OR = 1.3, CI = 0.95–1.9) *p* = 0.09
Gathirua-Mwangi 2016, USA [46]	African American women, different income levels Int DVD: *n* = 87 Int phone: *n* = 85 Cnt: *n* = 72 Age: 41–65	2 tailored interventions framed by the Health Belief and Transtheoretical Models: 1) perceived and actual risk; 2) perceived benefits, 3) barriers, 4) self-efficacy; and 5) knowledge Int DVD: Tailored, narrative DVD (delivered by an African American woman): Tailoring done by asking women to respond to questions about each of the beliefs plus provide demographic info. DVD showed animation of breast cancer developing and metastasis, video of mammography process, women were queried and received messages about individual barriers that would prevent them from receiving a mammogram, narrator encouraged viewers to make an appointment. (Intervention cost $6.84/person) Int phone: Computer-tailored telephone counselling (delivered by trained, graduate student counsellors: developed using the same tailoring variables. Messages used in the interactive DVD and the telephone intervention were kept consistent, so intervention content varied only by media delivery	Usual care: standard care from their healthcare providers	Mammography, self-report & medical records Follow-up: 6 months Int DVD: 41% Int phone: 42% Cnt: 35%, *p* = 0.6491 Neither the DVD nor phone had significant effects for women with household incomes > $30,000 For women with incomes ≤ $30,000 Int DVD vs Cnt: OR = 5.3, 95% CI = 1.1–25.4 Int phone vs Cnt: OR = 3.9, 95% CI = 0.8–18.5
Kreuter 2010, USA [23]^d^	African American women from low-income neighbourhoods Stratified by education and other variables Narrative Int: *n* = 244 Informative Int: *n* = 245 Mean age: 61.1	Two video interventions with 11 messages about breast cancer: breast cancer risk (you can get breast cancer at any age; you could have breast cancer without knowing; you could be at risk even if you have no family history), talking about breast cancer (learn your family history; talk openly about breast cancer; share breast cancer experiences; women can survive breast cancer), and getting a mammogram (mammograms can find breast lumps before you can feel them; get a mammogram yearly or every 1–2 years; mammograms can be uncomfortable but are not really painful; mammograms can save lives by finding breast lumps early) Narrative video “Living Proof” provided information in the form of personal stories from African American breast cancer survivors	Informative video, “Facts for Life” provided information in didactic, expository form from an African American woman narrator	Self-reported mammography Follow-up: 3 and 6 months Narrative Int: 48.6% vs Informative Int: 40%, *p* = 0.20 Women < 12 yrs. education Narrative Int: 64.5% vs Informative Int: 31.7%, *p* < 0.01 Women ≥ 12 yrs. education Narrative Int: 42.1% vs Informative Int: 43.1%, *p* = 0.91 Significant effect in women without a close family/friend with breast cancer but not after stratifying by income, age etc
Wang 2012, USA [47]	Chinese American immigrants Cnt: *n* = 222 Int1: *n* = 225 Int2: *n* = 217 Age: 50–70	Int1: Women viewed a video designed to debunk Chinese women’s culturally based beliefs about breast cancer and attitudes toward regular mammograms Int2: Women viewed a video targeting common issues on mammography use across different racial/ethnic groups, including knowledge, beliefs (e.g., fatalism), perceived barriers to care, and perceived risk for breast cancer	Chinese breast cancer fact sheet sent by mail	Mammography completion Follow-up: 12 months Acculturation was dichotomized on the basis of English ability and years of US residency Int1: 40.3% vs Int 2: 38.5% vs Cnt 31.1.%, *p* = 0.01 Low-acculturation OR = 1.7, CI = 1.04–2 High-acculturation OR = 1.2, CI = 0.6–2.7

BCT = behavioural construct tailoring; CI = 95% confidence interval; Cnt = control group; CRT = culturally relevant tailoring; Int = Intervention group; OR = odds ratio: RR = relative risk

### Colorectal Cancer (CRC) Screening

Nine studies reported findings from RCTS on CRC screening [31–39] (Table 5), of which two [38, 39] had data from the same trial. Several studies reported no effect of their interventions. In Davis et al.’s (14) study on low literacy, low-income, ethnically diverse communities, multicomponent, targeted, low-literacy materials were not found to be significantly different or more effective in increasing FIT uptake compared with the nontargeted materials. They instead suggested that the provision of a FIT test plus education may provide a key driver to improve CRC screening. Fernández et al. (15) evaluated a small media intervention consisting of a flipchart and DVD about CRC and screening compared to a tailored interactive multimedia intervention and usual care control. Neither of the two interventions increased screening uptake compared to controls. Gwede et al.’s (13) “LCARES” intervention featuring a Spanish language, low-literacy, culturally targeted photonovella booklet and DVD did not increase screening uptake compared to a standard Spanish-language booklet. Colonoscopy completion was also not significantly higher in Hoffman et al.’s (12) decision-aid video in an African American population. However, it did increase patients´ knowledge and reduced their decisional conflict. The only study outside of the US evaluated an intervention in Maori and Pacific people in New Zealand [32]. The DVD providing culturally tailored information on bowel cancer and FOBT included a famous Māori rugby player, who delivered key program messages aimed at improving knowledge and reducing barriers, including the ease and cleanliness of the test, and key features of invitation and program participation. The DVD also featured two well-known local Māori elders presenting a narrative description of their program participation experience. Surprisingly, FOBT screening was significantly lower in the intervention (13.6%) versus usual care controls (25.9%). However, spoiled kit rates were significantly higher among those who were not sent the DVD (33.1% versus 12.4% in Māori and 42.1% versus 21.9% in Pacific).

**Table 5:** Colorectal cancer (CRC) screening: Study characteristics and results of CRC screening including colonoscopy and Fecal Occult Blood Test (FOBT) / Fecal Immunochemical Test (FIT) (*n* = 9)

Author Year Country	Population N (Int, Cnt) Age range	Intervention (Int) description	Control (Cnt)	Results: Screening completion
Aragones 2010, USA [51]	Pairs of primary care physicians and Spanish-speaking Latino immigrants Int: *n* = 31 Cnt: *n* = 34 Age: > = 50	CRC educational video in Spanish on a portable personal digital video display device accompanied by a brochure with key information for the patient, and a patient-delivered paper-based reminder for their physician	Usual care	CRC screening completion (any) Follow-up: 3 months Int: 55% vs Cnt: 18%, *p* = 0.002 OR = 5.4, CI = 1.6–18.5
Bartholomew 2019, New Zealand [32]	Maori and Pacific people in New Zealand Int: *n* = 2388 Cnt: *n* = 2883 Age: > 50	A 6-min DVD providing culturally tailored information on bowel cancer and FOBT was sent to persons in the Maori and Pacific community who did not respond to the first call-to-screening A famous Māori rugby player delivered key messages aimed at improving knowledge and reducing barriers, including the ease and cleanliness of the test, and key features of invitation and program participation. The DVD also featured two well-known local Māori elders presenting a narrative description of their program participation experience	Usual care	CRC Screening with FOBT Follow-up: 4-weeks Int: 13.6% vs. Cnt: 25.9%, *p* = 0.011
Cameron 2011, USA [33]	Black: 25%, Uninsured or on Medicare or Medicaid: 22–24.9% Int: *n* = 314 Cnt: *n* = 314 Age: 50–79	A mailing consisting of a personalized reminder letter from the physician, an educational brochure, and a digital video disc (DVD) about CRC and CRC screening In addition, patients were called 2 weeks following the mailing to complete a brief process evaluation The DVD (“Get Screened for Colorectal Cancer Patient Education Program”) was designed based on the Extended Parallel Process Model of health behaviour change. Included common myths and questions regarding CRC and screening, info on FOBT and colonoscopy	Usual care	Screening rates at 3 months Int: 9.9% vs Cnt: 3.2% Rate ratio = 3.1; 95%CI = 1.5–6.2; *p* = 0.001 Screening rates at 6 months Int: 18.2% vs Cnt: 12.1% Rate ratio = 1.5, 95%CI = 1.03–2.2; *p* = 0.03
Davis 2017, USA [13]	Low literacy, low-income communities Ethnically diverse: 66% white, 10% Hispanic, 28% African American Int: *n* = 210 Cnt: *n* = 207 Age: 50–75	The CARES condition featured a targeted, low literacy, photonovella booklet and DVD informed by the constructs of the Preventive Health Model (e.g., salience, self-efficacy). The photonovella/DVD included storylines depicting local characters that modelled the test-specific behaviour of screening with the FIT kit. The photonovella/DVD content, storyline, photos, and graphics were informed by their prior work	Standard trifold CRC screening brochure developed by the Centres for Disease Control	FIT completion as reported by medical records Follow-up: 180 days Int: 78.1% vs Cnt: 83.5%, *p* = 0.17
Fernández 2015, USA [14]	Hispanics on the Texas–Mexico border TIMI Int: *n* = 236 SMPI Int: *n* = 236 Cnt: *n* = 204 Age:50–70 +	SMPI Int: Small Media intervention (SMPI): flipchart and DVD about CRC and CRCS TIMI Int: Tailored interactive multimedia intervention (TIMI) delivered using tablet computers with touch screen. Tailoring elements based on responses to questions about readiness (stage of change) to be screened, pros and cons, self-efficacy, perceived risk, and subjective norms. Intervention efforts focused on individuals in pre-contemplation (no CRCS and no intention), contemplation (no CRCS, but considering getting screened), and preparation (no CRCS and planning to get screened) stages of change. As participants proceeded through the interactive media, they were presented with various questions related to psychosocial factors. Based on responses, they were presented with information to address their particular concern or encourage screening based on current stage of readiness	No intervention	Any CRCS uptake 6 months Intention to treat analysis TIMI Int:10.2% SMPI Int: 13.6% Cnt: 10.8% Adjusted *p* = 0.46 No significant difference between groups
Gwede 2019, USA [12]	Latinos Int: *n* = 40 Cnt: *n* = 36 Age: 50–74	The LCARES intervention featured a Spanish language, low-literacy, culturally targeted photonovella booklet and DVD titled, ‘Un examen sencillo para un colon saludable’—a simple test for a healthy colon— informed by the constructs of the Preventive Health Model (e.g., salience and coherence, cancer worry and self-efficacy) plus FIT kit. The photonovella included storylines depicting characters that modelled the test-specific behaviour of FIT screening. The photonovella /DVD content, linguistics storyline, photos and graphics were informed by an extensive formative phase which included a series of focus groups and iterative processes to produce the intervention	Standard Spanish-language booklet developed by the Centers for Disease Control, ‘Las preubas de deteccio´n de ca´ncer colorrectal salvan vidas,’ plus a FIT kit	FIT uptake assessed by medical records Follow-up: 90 days Int: 90% vs Cnt: 83%, *p* = 0.379
Hoffman 2017, USA [11]	African American Int: *n* = 59 Cnt: *n* = 30 Age: 49–75	Entertainment-education, decision-aid video consisting of anatomy of the digestive system and colon, how colorectal cancer forms, who is at high risk of developing it, and morbidity/mortality rates. how colorectal cancer can potentially be prevented if polyps are detected and removed Three screening options (colonoscopy, FOBT, and sigmoidoscopy) were compared with respect to how each test works, how it is performed, preparations required by the patient, accuracy, recommended frequency, and other pros and cons. The Edutainment Decision Aid Model was used to improve saliency for African Americans and to ensure that the decision aid was accessible across literacy levels – it intersperses educational and decision support content, including tailored soap opera–like scenes of individuals modelling decision making behaviours	Attention control video about hypertension that contained similarly detailed information	Colonoscopy completion Follow-up: 3 months Int: 21% vs Cnt 28%, *p* = 0.45
Lairson 2018, USA [38] Shokar 2016, USA [39]	Low-income Hispanic Int Video: *n* = 160 Int Video + promotora *n* = 159 Int Promotora: *n* = 148 Cnt: *n* = 317 Age: 50–75	ACCION program: community-wide service and research program designed to educate and facilitate colorectal cancer screening compliance among low-income, uninsured Hispanic population The educational materials and intervention components were culturally tailored, addressed benefits (through provision of information about impact of screening on prevention, stage of diagnosis, mortality, and other benefits to family) Int Video: participants watched a motivational video with information about CRC and the importance of screening Int Video + Promotor: a promotora played the video and had specified pauses for standardized interactive activities Int Promotora: involved the use of a flip chart for explaining the same content covered in the video	No intervention	Self-reported screening uptake Follow-up: 6 months Int Video: 78% Int Promotora; 87.1% Int Video + Promotor: 83.2%, Cnt: 10.1% “Significantly higher” (p value not reported) Int (all): 80.5% vs Cnt: 17.0%, *p* < 0.001 RR = 4.7, CI = 3.7–6.1, *p* < 0.001

CI = 95% confidence interval; Cnt = control group; CRC = colorectal cancer; FIT = Faecal Immunochemical Test; FOBT = fecal occult blood test; Int = Intervention group; OR = odds ratio: RR = relative risk; SMPI = Small Media intervention

The other studies, however, did report some positive results. Aragones et al. [51] found higher CRC screening in Latino immigrants at three-month follow-up using an educational video in Spanish on a portable personal digital video display device accompanied by a brochure with key information for the patient, and a patient-delivered paper-based reminder for their physician, compared to a usual care control. Of note, the intervention’s success may have been due to the fact that it targeted both physicians (through a patient-delivered paper-based reminder for their physician) and patients. Cameron et al. [33] reported significantly higher screening at both three and six months in their intervention versus usual care control. The intervention was a mailing consisting of a personalized reminder letter from the physician, an educational brochure, and a DVD about colorectal cancer and colorectal cancer screening. Lairson et al.[38] and Shokar et al.[39] reported data from the same trial, the ACCION program: a community-wide service and research program designed to educate and facilitate colorectal cancer screening compliance among a low-income, uninsured Hispanic population. Interventions included a video-only arm, a promotora-only arm, and a video-plus promotora arm. The screening was higher in participants who only viewed the video compared to controls (78% vs 10.1%).

### Risk of Bias

Random sequence generation (selection bias): Seven studies had a low risk of bias. The authors described in detail a random component of the sequence-generation process. Eight studies were assessed as having an unclear risk of bias, as no information about the randomization process was provided. One study had a high risk of bias because the investigators described a non-random component in the sequence generation process.

Allocation concealment (selection bias): Two studies were judged at low risk of bias, as the allocation methods used were appropriate. Two studies had a high risk of bias because investigators enrolling participants could possibly foresee assignments. Twelve studies were assessed with an unclear risk of bias as they contained no information about allocation concealment procedures.

Blinding of participants and personnel (performance bias): In four studies unlikely that the blinding could have been broken, so the risk of bias was judged as low. Ten studies were assessed with a high risk, due to lack of blinding or incomplete blinding. Two studies were judged with an unclear risk, due to lack of information about blinding of participants and providers.

Blinding of outcome assessment (detection bias): In three studies the outcome assessment was blinded, so the risk of bias was judged as low. Eight studies were assessed with a high risk, as the outcome assessment was not blinded. Five studies were judged with an unclear risk, due to lack of information about blinding of outcome assessor.

Incomplete outcome data (attrition bias): Ten studies were assessed with a low risk of bias because no missing data were found, or the purpose of participants’ exclusion was properly argued. No studies had a high risk of bias related to the number of drop-outs due to missing primary outcome data. Six studies were judged with an unclear risk, due to lack of information about a reason for missing data.

**Fig. 2:**
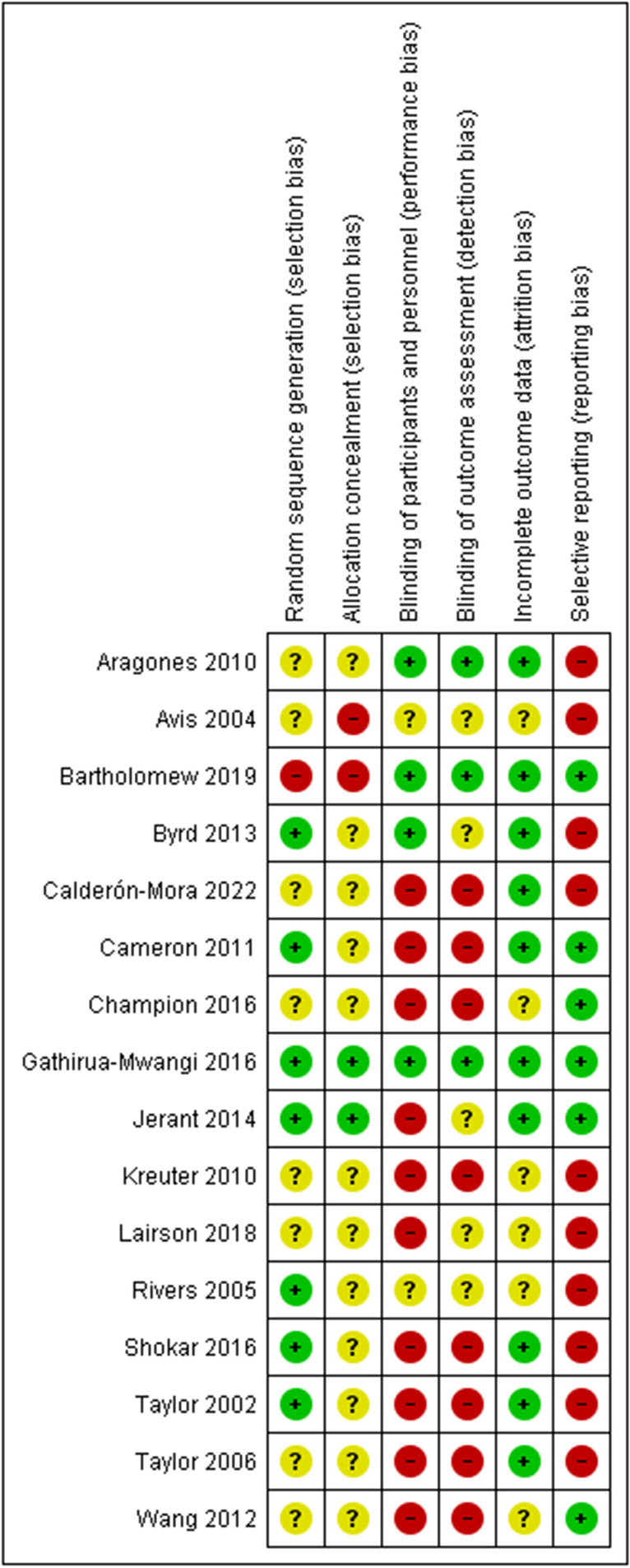
Risk of bias summary: review authors’ judgements about each risk of bias item for each included study

**Fig. 3:**
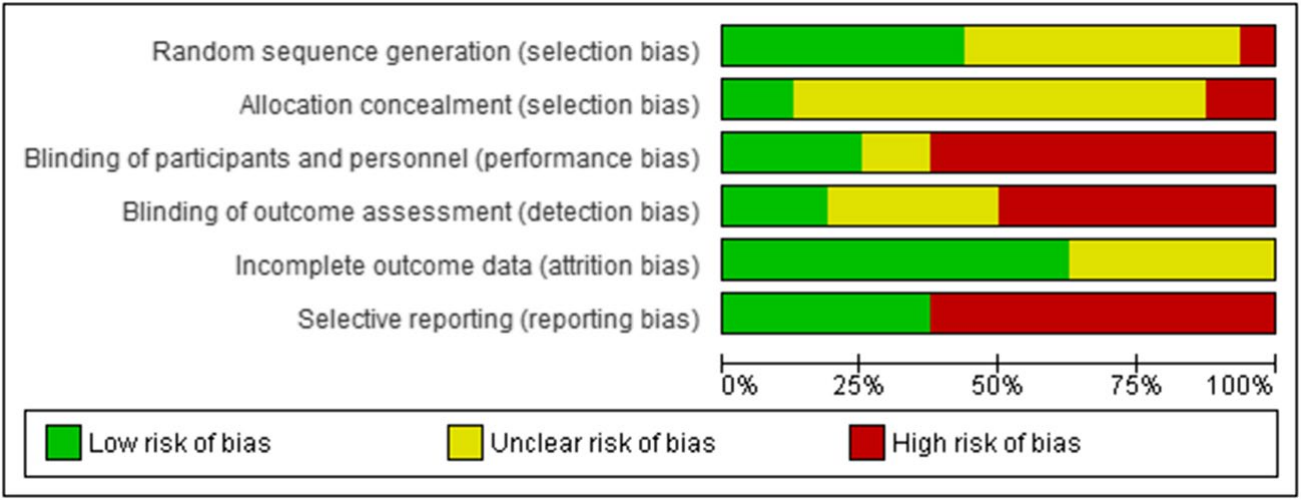
Risk of bias graph: review authors’ judgements about each risk of bias item presented as percentages across all included studies

Selective reporting (reporting bias): Six studies were judged with a low risk of bias because the study protocol was registered with the study’s pre-specified outcomes. Study protocol was not available for ten studies and, thus, they were judged with a high risk. Detailed risk of bias in the included studies is shown in Fig. 2, whereas the overall quality of included studies can be observed in Fig. 3.

## Discussion

### Main Findings

Our systematic review identified twenty studies that assessed video interventions aimed to increase cancer screening in ethnic and racial minority groups, and populations with low income. For cervical cancer screening, most of the video interventions reported positive results. Studies aimed at increasing mammography uptake generally were only effective in specific groups of participants, such as low-income or less-educated African American women. Studies on CRC screening reported conflicting results, with half finding significant effects on screening uptake and the rest reporting no difference in screening between intervention and control participants. Of note, except for one study, all were based in the USA; thus, the current scientific evidence cannot be generalized to other countries.

### Differences in Intervention Effects According to the Characteristics of the Participant

Overall, the results of video-based interventions had varying effects in terms of improving screening uptake compared to usual care conditions. Generally, interventions that included culturally and ethically tailored videos were more effective. Importantly, the effects of some interventions differed according to the characteristics of the individual. Champion et al.[49] and Gathirua-Mwangi et al.[46] reported significant effects on mammography screening of their tailored DVD interventions only in low-income African American women, but not in those with higher incomes. Similarly, Kreuter et al. [23] reported effects only in African American women with lower education. Latina women age 51–65 years were the only age group to have a significant difference in screening uptake for cervical cancer in a novella-style video intervention [41]. Wang et al. [7] also only found significant effects of a culturally targeted versus generic video only among low-acculturated Chinese American women, whereas the two videos did not lead to different screening uptake in high-acculturated women. They discussed that recently immigrated women and ones with limited English language abilities find it challenging to assimilate to the US cultural environment and face challenges such as access to care. Their culturally targeted video included an all-Chinese cast and many Chinese cultural features that may have helped low-acculturated women find it easier to relate to the video and, thus, their level of involvement increases. These results are promising, as they suggest that tailoring the content to the target audience may create effective strategies to help specific medically underserved populations. A meta-analysis concluded that mammography attendance is generally lower among immigrant and minority women compared to other women (46.2% vs 55.0%; odds ratio = 0.64) [52] and lack of knowledge is thought to be a key barrier to attending breast screening in Black, Asian and Minority Ethnic (BAME) women [53] but designing studies of barriers around race and ethnicity is not always appropriate because other demographic factors may play a role [54]. Gathirua-Mwangi et al. [46] reported that the low-income participants in their study paid significantly more attention to the DVD than higher-income women and, thus, their intervention may have been more successful in this group because the increased attention could maximize their learning. They further hypothesized that the narrative format of the DVD may also have played a role. Champion et al. [49] described that their DVD better impacted lower-income participants because the majority of female actors in the film were cast as women with lower incomes but also postulated that the reduced effect of the intervention in higher-income women may be due to DVDs being less engaging format for health education in this group than the internet, for example.

### The Potential use of Video in Media and Social Media Campaigns

In the current review, we included RCTs that focused on videos and DVDs as education and training tools to increase knowledge and improve health literacy compared to usual care. However, these studies were conducted in rigorously controlled research conditions, and it may be of interest to see how they can be applied in more realistic settings. Specifically, they may have more far-reaching effects if used within a large media education campaign. In the “REACHing Vietnamese American Women: A Community Model for Promoting Cervical Cancer Screening (REACH)” study [55] used a media education campaign and a lay health worker outreach program to increase Vietnamese American women’s cervical cancer awareness, knowledge, and screening. They used Vietnamese-language television channels to broadcast television ads, along with radio and newspaper ads and concluded that media education campaigns can increase Vietnamese women’s awareness of the importance of pap tests, although it was higher when the intervention was combined with a lay health worker, the media campaign alone did increase pap test uptake. Due to the increasing use of social media, and its potential usage in delivering health education, it may be of interest to assess ways of adapting video interventions for use in social media, though this field is still relatively new. A review of social media and mHealth technologies for cancer screening found only four studies with social media interventions [56]. Some of the benefits of using social media for health communication include increased interactions with others and more available, shared, and tailored information, as well as peer/social/emotional support [57]. Further, adapting videos to social media platforms may be an important way of targeting hard-to-reach populations. For example, the use of social media and videos were recommended communication channels for breast cancer educational messages for young African American women in a study using informant interviews [57]. These women face health inequities that place them at greater risk for mortality from breast cancer [58]. Another study reported that a large proportion of medically underserved women are overdue for cervical cancer screening, but they regularly use social media and are willing to participate in social media-driven interventions [50].

### Advantages of Video and DVD Interventions for Screening Promotion

There are several advantages to delivering health inequity interventions via video and DVD. First, delivery can be modified according to the characteristics of the individual, for example in different languages or with differing material according to age or health literacy levels. Second, they might be cost-effective in terms of reaching large amounts of people. Cost-effectiveness was not investigated here, as it was not the primary aim of our review, although it is of great relevance. Gathirua-Mwangi et al.´s [46] article reported that the DVD arm of their study was three times less expensive than a telephone intervention. In the ACCION program Lairson et al. [38] reported that, when delivered to a group, their video was the most cost-effective CRC screening promotion intervention, compared to other study arms that included a promotora. A further advantage of video and DVD interventions is that they have the potential to be delivered remotely, for example, several trials mailed DVDs and videos to participants [33, 47, 49]. This is of growing importance due to the COVID-19 pandemic, when worldwide screening rates for breast, colon, and cervical cancer were lower [28], and health inequities may be increased; for example, there was a lower likelihood of returning for breast cancer screening after COVID-19-related closures for people in higher poverty areas, those without health insurance, people who need an interpreter, and those with longer travel times [59]. Delivering interventions to promote cancer screening remotely can help to target specific groups during periods when public health restrictions are tightened and there are changes to routine medical services and a reduction in face-to-face health promotion programs.

### Strengths and Limitations

The strength of the current study is the systematic, comprehensive literature search with thorough study selection and quality assessment. However, some limitations should be noted. We only found one study outside of the US and, thus, it may be difficult to generalize any findings to other settings, especially as the US has no universal healthcare coverage and has specific health inequity challenges associated with this. We also only reported studies in the literature that focused on cultural, ethnic, or economic inequity. It may be of interest to conduct a future systematic review specifically on interventions that target other groups that face cancer screening inequity such as incarcerated women [60] or LGBTQ persons [61]. It is also worth discussing that we only included RCTs that had a video-only intervention arm, as we wanted to focus on how tailoring the delivery may affect screening behaviour. Videos are often used in conjunction with other health promotion components such as group or individual health education and counselling, such as Byrd et al.‘s [40] study, which did not find differences in cervical cancer screening in participants in the video-only arm of their study compared to a more complex intervention arm that included training by a promotora, the instructional video, and a flip chart. Similar results were also reported for FIT uptake in a USA study [62]. So et al. [63] used an interesting approach that involved targeting older South Asian adults together with one of their younger family members in Hong Kong, which included culturally and linguistically relevant video clip, but this was used in conjunction with other components such as an instructor-led health presentation and health information booklet [63]. Although it was successful in increasing FIT screening uptake, it was not possible to isolate the effect of the video component as the intervention had multiple components. Further, we only included studies that had screening completion as an outcome, although there are studies that look instead at other related outcomes such as knowledge and attitudes regarding screening or intention to participate in screening in the future. For example, a study in the Netherlands reported that a culturally sensitive educational video targeting Turkish and Moroccan women resulted in more positive screening attitudes compared to the normal information brochure [64] but such studies were not included as we aimed to focus on concrete screening behaviours. Finally, it is important to consider that these trials include small, specific groups of people, and it is not clear whether they can actually lead to improvements in health equity from a larger perspective. These interventions need to be viewed from a broader standpoint that considers the wealth of changes needed to achieve a meaningful shift in equity, from a Health in All Policies approach that requires action from multiple sectors.

### Future Research

It will be of interest to identify effective intervention strategies within European settings and other countries worldwide to assess differences in screening barriers and uptake between countries and whether these need different interventions and modalities to target them. Studies that can adapt already existing video-based interventions to other groups with high health inequity may also be relevant. More research is needed on specific subgroups, for example, young minority women, who face health inequities that place them at greater risk for mortality from breast cancer [58]. As discussed above, the cost-effectiveness of interventions is important, and all future trials should include a measurement of cost in addition to screening completion as an outcome. It is worth noting that culturally tailored video interventions have also been shown to increase illness knowledge in specific groups of people with cancer; for example, a pilot study in Amazonian women in treatment for cervical cancer reported increased knowledge about their illness. Thus, it will be of interest to extend studies on specific groups such as these to examine whether video interventions can be adapted also for the purpose of increasing screening uptake.

## Conclusions

In conclusion, although results are mixed, video interventions to promote screening for breast, cervical, and colorectal cancer in this field have some positive results, especially if they are tailored. During and after the COVID-19 pandemic, further testing and development of effective intervention strategies that can be delivered remotely, such as videos, may provide relevant health promotion strategies that can help to reduce health inequities in cancer screening.

## Appendix 1

Table

**Table 6:** PubMed and Cochrane search terms

PubMed
("neoplasms"[MeSH Terms] OR Neoplas* OR Tumor* Or Cancer* OR Malignan* OR "Malignant Neoplasm*" OR "Neoplasm, Malignant") AND ("diagnosis"[Subheading] OR "mass screening"[MeSH Terms] OR "early detection of cancer"[MeSH Terms] OR Screening[Text Word]) AND ("health equity" OR "health inequity" OR "health inequality" OR "health equality" OR "health disparities" OR inequity OR equity OR race OR racial OR socioeconomic OR SES OR income OR minority OR latin*)
Cochrane
(('neoplasms' OR 'neoplasms' OR neoplas* OR tumor* OR cancer* OR malignan* OR 'malignant neoplasm*')):ti,ab,kw AND (('diagnosis' OR 'mass screening' OR 'early detection of cancer' OR screening)):ti,ab,kw AND (('health equity' OR 'health inequity' OR 'health inequality' OR 'health equality' OR 'health disparities' OR inequity OR equity OR race OR racial OR socioeconomic OR ses OR income OR minority OR latin*)):ti,ab,kw AND ((randomised OR randomized OR randomisation OR randomisation OR random*)):ti,ab,kw

6

## Appendix 2

Risk of bias tables—characteristics of included studies.

Table

**Table 7:** Risk of bias for Aragones et al., 2010.* Aragones A, Schwartz MD, Shah NR, Gany FM. A randomized controlled trial of a multilevel intervention to increase colorectal cancer screening among Latino immigrants in a primary care facility. J Gen Intern Med. 2010;25(6):564–7*

Bias	Authors' judgement	Support for judgement
Random sequence generation (selection bias)	Unclear risk	Insufficient information
Allocation concealment (selection bias)	Unclear risk	Insufficient information
Blinding of participants and personnel (performance bias)	Low risk	Patients were blind to their physician’s randomization
Blinding of outcome assessment (detection bias)	Low risk	A research assistant, not involved in patient recruitment and blind to the randomization assignment, reviewed electronic medical records 3 months after the index visit to determine the primary outcome
Incomplete outcome data (attrition bias)	Low risk	No missing data
Selective reporting (reporting bias)	High risk	The study protocol is not available

7

Table

**Table 8:** Risk of bias for Avis et al., 2004. *Avis NE, Smith KW, Link CL, Goldman MB. Increasing mammography screening among women over age 50 with a videotape intervention. Prev. Med. 2004;39(3):498–506*

Bias	Authors' judgement	Support for judgement
Random sequence generation (selection bias)		
Allocation concealment (selection bias)		
Blinding of participants and personnel (performance bias)		
Blinding of outcome assessment (detection bias)		
Incomplete outcome data (attrition bias)		
Selective reporting (reporting bias)		

8

Table

**Table 9:** Risk of bias for Bartholomew et al., 2019. *Bartholomew K, Zhou L, Crengle S, Buswell E, Buckley A, Sandiford P. A targeted promotional DVD fails to improve Māori and Pacific participation rates in the New Zealand bowel screening pilot: results from a pseudo-randomised controlled trial. BMC Public Health. 2019;19(1):1245*

Bias	Authors' judgement	Support for judgement
Random sequence generation (selection bias)	High risk	The investigators describe a non-random component in the sequence generation process
Allocation concealment (selection bias)	High risk	The investigators describe a non-random component in the sequence generation process
Blinding of participants and personnel (performance bias)	Low risk	Participants and those recording the outcomes (receipt of a spoiled or non-spoiled test kit) were blinded to group assignment
Blinding of outcome assessment (detection bias)	Low risk	Participants and those recording the outcomes (receipt of a spoiled or non-spoiled test kit) were blinded to group assignment
Incomplete outcome data (attrition bias)	Low risk	No missing outcome data
Selective reporting (reporting bias)	Low risk	The study protocol is available

9

Table

**Table 10:** Risk of bias for Byrd et al., 2013. *Byrd TL, Wilson KM, Smith JL, Coronado G, Vernon SW, Fernandez-Esquer ME, *et al*. AMIGAS: a multicity, multicomponent cervical cancer prevention trial among Mexican American women. Cancer. 2013;119(7):1365–72. *https://doi.org/10.1002/cncr.27926

Bias	Authors' judgement	Support for judgement
Random sequence generation (selection bias)	Low risk	Computer-generated randomization scheme to randomly assign 613 women to1 of 4 study arms that differed by the types of materials the promotoras used to deliver the program
Allocation concealment (selection bias)	Unclear risk	No information
Blinding of participants and personnel (performance bias)	Low risk	No blinding of outcome assessment, but the review authors judge that the outcome measurement is not likely to be influenced by lack of blinding
Blinding of outcome assessment (detection bias)	Unclear risk	Insufficient information to permit judgement
Incomplete outcome data (attrition bias)	Low risk	No missing outcome data
Selective reporting (reporting bias)	High risk	The study protocol is not available

10

Table

**Table 11:** Risk of bias for Calderón-Mora et al., 2022. *Calderón-Mora J, Alomari A, Shokar N. Comparison of Narrative Video and Flipchart Presentation to Promote Cervical Cancer Screening Among Latinas Along the Border. Health Educ. Behav. 2022:10,901,981,221,074,918*

Bias	Authors' judgement	Support for judgement
Random sequence generation (selection bias)	Unclear risk	Insufficient information about the sequence generation process
Allocation concealment (selection bias)	Unclear risk	Insufficient information to permit judgement
Blinding of participants and personnel (performance bias)	High risk	No blinding described
Blinding of outcome assessment (detection bias)	High risk	No blinding of outcome assessment described
Incomplete outcome data (attrition bias)	Low risk	No missing outcome data
Selective reporting (reporting bias)	High risk	The study protocol is not available

11

Table

**Table 12:** Risk of bias for Cameron et al., 2011. *Cameron KA, Persell SD, Brown T, Thompson J, Baker DW. Patient outreach to promote colorectal cancer screening among patients with an expired order for colonoscopy: a randomized controlled trial. Arch Intern Med. 2011;171(7):642–6*

Bias	Authors' judgement	Support for judgement
Random sequence generation (selection bias)	Low risk	Authors used a random number generator
Allocation concealment (selection bias)	Unclear risk	Insufficient information
Blinding of participants and personnel (performance bias)	High risk	No blinding
Blinding of outcome assessment (detection bias)	High risk	No blinding of outcome assessment
Incomplete outcome data (attrition bias)	Low risk	No missing outcome data
Selective reporting (reporting bias)	Low risk	The study protocol available

12

Table

**Table 13:** Risk of bias for Champion et al., 2016. *Champion VL, Rawl SM, Bourff SA, Champion KM, Smith LG, Buchanan AH, *et al*. Randomized trial of DVD, telephone, and usual care for increasing mammography adherence. J Health Psychol. 2016;21(6):916–26*

Bias	Authors' judgement	Support for judgement
Random sequence generation (selection bias)	Unclear risk	Insufficient information about sequence generation
Allocation concealment (selection bias)	Unclear risk	Insufficient information
Blinding of participants and personnel (performance bias)	High risk	No blinding
Blinding of outcome assessment (detection bias)	High risk	No blinding of outcome assessment
Incomplete outcome data (attrition bias)	Unclear risk	No reasons for missing data provided
Selective reporting (reporting bias)	Low risk	The study protocol is available

13

Table

**Table 14:** Risk of bias for Gathirua-Mwangi et al., 2016. *Gathirua-Mwangi WG, Monahan PO, Stump T, Rawl SM, Skinner CS, Champion VL. Mammography Adherence in African American Women: Results of a Randomized Controlled Trial. Ann Behav Med. 2016;50(1):70–8*

Bias	Authors' judgement	Support for judgement
Random sequence generation (selection bias)	Low risk	Simple random assignment to the three groups was performed with a computer using a random number generator
Allocation concealment (selection bias)	Low risk	The allocation assignment was concealed by the computer program until interventions were assigned
Blinding of participants and personnel (performance bias)	Low risk	The investigators generated the random allocation sequence,
Blinding of outcome assessment (detection bias)	Low risk	Blinding of outcome assessment ensured
Incomplete outcome data (attrition bias)	Low risk	Missing outcome data balanced in numbers across intervention groups
Selective reporting (reporting bias)	Low risk	The study protocol is available

14

Table

**Table 15:** Risk of bias for Jerant et al., 2014. Jerant A, Kravitz RL, Sohler N, Fiscella K, Romero RL, Parnes B, et al. Sociopsychological tailoring to address colorectal cancer screening disparities: a randomized controlled trial. Ann Fam Med. 2014;12(3):204–14

Bias	Authors' judgement	Support for judgement
Random sequence generation (selection bias)	Low risk	Patients were randomly assigned by the software using a random number generation program
Allocation concealment (selection bias)	Low risk	Investigators enrolling participants could not foresee assignment because of randomization
Blinding of participants and personnel (performance bias)	High risk	Data collection personnel were not alerted to participants’ study group
Blinding of outcome assessment (detection bias)	Unclear risk	Insufficient information
Incomplete outcome data (attrition bias)	Low risk	Missing outcome data balanced in numbers across intervention groups
Selective reporting (reporting bias)	Low risk	The study protocol is available with study’s pre-specified outcomes

15

Table

**Table 16:** Risk of bias for Kreuter et al., 2010. Kreuter MW, Holmes K, Alcaraz K, Kalesan B, Rath S, Richert M, et al. Comparing narrative and informational videos to increase mammography in low-income African American women. Patient Education and Counselling. 2010;81(SUPPL.1): S6-S14

Bias	Authors' judgement	Support for judgement
Random sequence generation (selection bias)	Unclear risk	The investigators describe a random component but not the sequence generation process
Allocation concealment (selection bias)	Unclear risk	Concealment is not described
Blinding of participants and personnel (performance bias)	High risk	No blinding described and the outcome can be influenced by lack of blinding
Blinding of outcome assessment (detection bias)	High risk	No blinding of outcome assessment
Incomplete outcome data (attrition bias)	Unclear risk	Insufficient information to permit judgement
Selective reporting (reporting bias)	High risk	The study protocol is not available

16

Table

**Table 17:** Risk of bias for Lairson et al., 2018. Lairson DR, Kim J, Byrd T, Salaiz R, Shokar NK. Cost-Effectiveness of Community Interventions for Colorectal Cancer Screening: Low-Income Hispanic Population. Health Promot. Pract. 2018;19(6):863–72

Bias	Authors' judgement	Support for judgement
Random sequence generation (selection bias)	Unclear risk	The investigators describe a random component but not the sequence generation process
Allocation concealment (selection bias)	Unclear risk	Concealment is not described
Blinding of participants and personnel (performance bias)	High risk	No blinding
Blinding of outcome assessment (detection bias)	Unclear risk	Insufficient information
Incomplete outcome data (attrition bias)	Unclear risk	Insufficient information
Selective reporting (reporting bias)	High risk	The study protocol is not available

17

Table

**Table 18:** Risk of bias for Rivers et al., 2005. Rivers SE, Salovey P, Pizarro DA, Pizarro J, Schneider TR. Message framing and pap test utilization among women attending a community health clinic. J Health Psychol. 2005;10(1):65–77

Bias	Authors' judgement	Support for judgement
Random sequence generation (selection bias)	Low risk	Groups of participants were assigned to frame and behaviour function conditions using a computer-generated table of randomly sorted combinations of conditions
Allocation concealment (selection bias)	Unclear risk	Insufficient information
Blinding of participants and personnel (performance bias)	Unclear risk	Insufficient information
Blinding of outcome assessment (detection bias)	Unclear risk	Insufficient information
Incomplete outcome data (attrition bias)	Unclear risk	Insufficient information
Selective reporting (reporting bias)	High risk	The study protocol is not available

18

Table

**Table 19:** Risk of bias for Shokar et al., 2016. Shokar NK, Byrd T, Salaiz R, Flores S, Chaparro M, Calderon-Mora J, et al. Against colorectal cancer in our neighbourhoods (ACCION): A comprehensive community-wide colorectal cancer screening intervention for the uninsured in a predominantly Hispanic community. Prev. Med. 2016;91:273–80. d

Bias	Authors' judgement	Support for judgement
Random sequence generation (selection bias)	Low risk	Study participants were randomly allocated to experimental or control arm using a computerized randomly generated block sequence
Allocation concealment (selection bias)	Unclear risk	Insufficient information
Blinding of participants and personnel (performance bias)	High risk	No blinding
Blinding of outcome assessment (detection bias)	High risk	No blinding of outcome assessment
Incomplete outcome data (attrition bias)	Low risk	Missing outcome data balanced in numbers across intervention groups
Selective reporting (reporting bias)	High risk	The study protocol is not available

19

Table

**Table 20:** Risk of bias for Taylor et al., 2002. Taylor VM, Hislop TG, Jackson JC, Tu SP, Yasui Y, Schwartz SM, et al. A randomized controlled trial of interventions to promote cervical cancer screening among Chinese women in North America. J Natl Cancer Inst. 2002;94(9):670–7

Bias	Authors' judgement	Support for judgement
Random sequence generation (selection bias)	Low risk	A computer program was used to randomly allocate each woman to one of the three study arms
Allocation concealment (selection bias)	Unclear risk	Insufficient information
Blinding of participants and personnel (performance bias)	High risk	No blinding
Blinding of outcome assessment (detection bias)	High risk	No blinding of outcome assessment
Incomplete outcome data (attrition bias)	Low risk	Missing data have been imputed using appropriate methods
Selective reporting (reporting bias)	High risk	The study protocol is not available

20

Table

**Table 21:** Risk of bias for Taylor et al., 2006. Taylor KL, Davis JL, 3rd, Turner RO, Johnson L, Schwartz MD, Kerner JF, et al. Educating African American men about the prostate cancer screening dilemma: a randomized intervention. Cancer Epidemiol Biomarkers Prev. 2006;15(11):2179–88

Bias	Authors' judgement	Support for judgement
Random sequence generation (selection bias)	Unclear risk	Insufficient information about the sequence generation process
Allocation concealment (selection bias)	Unclear risk	Insufficient information
Blinding of participants and personnel (performance bias)	High risk	No binding
Blinding of outcome assessment (detection bias)	High risk	No blinding of outcome assessment
Incomplete outcome data (attrition bias)	Low risk	Missing outcome data balanced in numbers across intervention groups
Selective reporting (reporting bias)	High risk	The study protocol is not available

21

Table

**Table 22:** Risk of bias for Wang et al., 2012. Wang JH, Schwartz MD, Brown RL, Maxwell AE, Lee MM, Adams IF, et al. Results of a randomized controlled trial testing the efficacy of a culturally targeted and a generic video on mammography screening among Chinese American immigrants. Cancer Epidemiol Biomarkers Prev. 2012;21(11):1923–32

Bias	Authors' judgement	Support for judgement
Random sequence generation (selection bias)	Unclear risk	Insufficient information about the sequence generation process
Allocation concealment (selection bias)	Unclear risk	Insufficient information
Blinding of participants and personnel (performance bias)	High risk	No binding
Blinding of outcome assessment (detection bias)	High risk	No blinding of outcome assessment
Incomplete outcome data (attrition bias)	Unclear risk	Insufficient information: number of drop out not reported for each group
Selective reporting (reporting bias)	Low risk	The study protocol is available

22
